# miR-19b promotes breast cancer metastasis through targeting MYLIP and its related cell adhesion molecules

**DOI:** 10.18632/oncotarget.19278

**Published:** 2017-07-17

**Authors:** Luqing Zhao, Yuelong Zhao, Yanong He, Yitao Mao

**Affiliations:** ^1^ Department of Pathology, Xiangya Hospital, Central South University, Changsha, Hunan 410008, China; ^2^ Department of Radiology, Xiangya Hospital, Central South University, Changsha, Hunan 410008, China; ^3^ School of Computer Science and Engineering, South China University of Technology, Guangzhou, Guangdong 510640, China; ^4^ Department of Pathology, School of Basic Medical Science, Xiangya School of Medicine, Central South University, Changsha, Hunan 410013, China

**Keywords:** miR-19b, MYLIP, cell adhesion molecules, breast cancer, metastasis

## Abstract

miR-19b is a key molecule for cancer development, however its crucial roles in breast cancer metastasis are rarely studied right now. In this study, using several bioinformatics databases to predict the downstream targets for miR-19b, we verified that a novel target gene, myosin regulatory light chain interacting protein (MYLIP), could be directly down-regulated by miR-19b through its 3′-UTR region. MYLIP belongs to the cytoskeletal protein clusters and is involved in the regulation of cell movement and migration. We further explored that miR-19b was highly expressed and negatively correlated with MYLIP expression in breast cancer patient samples from the TCGA database. And the over-expression of miR-19b or inhibition of MYLIP facilitated the migration and metastasis of breast cancer cells, through conducting the wound healing assay and transwell invasion assay. Additionally, miR-19b could obviously promote breast tumor growth in mouse models and affect the expressions of cell adhesion molecules (including E-Cadherin, ICAM-1 and Integrin β1) by down-regulating E-Cadherin expression and up-regulating ICAM-1 and Integrin β1 expressions *in vitro* and *in vivo*. Meanwhile, miR-19b effectively activated the Integrin β downstream signaling pathways (such as the Ras-MAPK pathway and the PI3K-AKT pathway) and elevated the expression levels of essential genes in these two pathways. Taken together, these findings comprehensively illustrate the regulatory mechanisms ofmiR-19b in breast cancer metastasis, and provide us new insights for exploring MYLIP and its related cell adhesion molecules as promising therapeutic targets to interfere breast cancer development.

## INTRODUCTION

Breast cancer is one of the most prevalent cancers occurred among women worldwide. Although the early diagnostic methods and therapeutic effects for this cancer are rapidly developed, its distant metastasis is still the main reason for patients death [1, 2]. So deeply exploring the mechanisms of preventing breast cancer metastasis are of great importance. miRNA is a cluster of key regulatory molecules involved in various processes of cancer development, including the cell growth, proliferation, cell cycle, apoptosis, migration, metastasis and chemo- or radio-resistance [3–5]. In the latest studies, for example, miR-182, miR-151-3p, miR-320a and miR-494 have been reported to exert their essential roles in modulating the metastasis of breast cancer cells through targeting SNAI1, TWIST1, MTDH, PAK1 and so on [6–9].

In our previous research, we found that miR-19b can be positively regulated by DEAD-box RNA helicase DDX3X and has significant expression difference in breast cancer [10]. Meanwhile, the recent publications have showed that miR-19b could suppress the expression level of protein tyrosine phosphatase receptor type G (PTPRG) so as to increase cell proliferation, reduce apoptosis and finally promote breast tumorigenesis [11]. Also, curcumin could modulate the miR-19/PTEN/AKT/p53 axis to exhibit its protective effects against bisphenol A (BPA)-induced breast cancer cell proliferation [12]. However, the potential roles of miR-19b in breast cancer metastasis are rarely studied and largely unknown until now, thus we launch this study for thoroughly discovering the regulatory mechanisms of miR-19b in the metastasis of breast cancer cells.

Using the bioinformatics databases to predict the downstream targets of miR-19b, we noticed that a novel target gene, myosin regulatory light chain interacting protein (MYLIP), has the high score ranks and might participate in the processes of cancer metastasis. MYLIP belongs to the cytoskeletal protein clusters and is involved in the regulation of cell movement and migration. It is a new member of ERM (ezrin, radixin, moesin) proteins family, and shares the similarity in the biological functions with ERM proteins [13, 14]. Through interacting the cell membrane proteins with myosin cytoskeleton, MYLIP plays a key role in the maintenance of cellular morphology, the modulation of cell motility, the remodeling of cytoskeletal proteins, and the adhesion of cells with extracellular matrix (ECM).

More importantly, the ERM family proteins have an important impact on the cell extension, growth, differentiation and migration via their intimately interactions with cell adhesion molecules, such as E-Cadherin, ICAM-1, ICAM-2, Integrins, CD44 and so on [15]. Once the protein expression levels or the gene mutations occurred in these cell adhesion molecules, the intercellular contacts and junctions will be changed and the cells become looser to spread out, so as to facilitate the migration, invasion and metastasis of tumor cells [16]. Nevertheless, there is no study focused on the significant role of MYLIP in cancer development and progression processes nowadays, so in this paper we will try to fully discuss the potential mechanisms of miR-19b regulating MYLIP and its related cell adhesion molecules expressions, and their biological functions in breast cancer metastasis, finally paving new avenues for searching useful therapeutic targets of breast cancer.

## RESULTS

### The expression of miR-19b is negatively correlated with MYLIP expression in breast cancer patient samples

**Figure 1:**
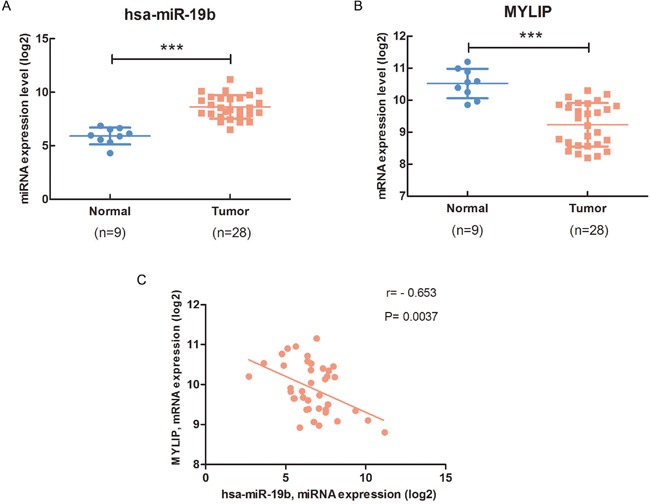
The expression of miR-19b is negatively correlated with MYLIP expression in breast cancer patient samples **(A)** The hsa-miR-19b expression level in breast cancer samples (n=28) and adjacent normal breast tissues (n=9). The primary hsa-miR-19b expression data (log2) in breast cancer patients were downloaded from the TCGA database. The asterisks (***) indicate a significant difference (*p* < 0.001). **(B)** The MYLIP expression level in breast cancer samples (n=28) and adjacent normal breast tissues (n=9). The primary MYLIP mRNA expression data (log2) in breast cancer patients were downloaded from the TCGA database. The asterisks (***) indicate a significant difference (*p* < 0.001). **(C)** The correlation between hsa-miR-19b and MYLIP in breast cancer samples (n=40). The primary hsa-miR-19b expression data (log2) and MYLIP mRNA expression data (log2) in breast cancer patients were downloaded from the TCGA database.

Firstly, we used the TCGA (The Cancer Genome Atlas) database to analyze the expression level of hsa-miR-19b in breast cancer samples and adjacent normal breast tissues. The data showed that hsa-miR-19b expression level was significantly up-regulated in breast cancer samples compared with normal breast tissues (***p < 0.001) (Figure 1A, Supplementary Figure 1A). Using the TargetScan, miRecords, miRanda, PITA and RNAhybrid databases, we predicted that MYLIP might be a putative downstream target of miR-19b. Then we adopted the primary MYLIP mRNA expression data (log2) in breast cancer samples and adjacent normal breast tissues from the TCGA database, and found that the expression level of MYLIP was significantly down-regulated in breast cancer samples compared with normal breast tissues (***p < 0.001) (Figure 1B, Supplementary Figure 1B). To further validate the inverse correlation between hsa-miR-19b and MYLIP, we conducted the correlation analysis to test the expression levels of hsa-miR-19b and MYLIP in breast cancer samples at the same time. The data indicated that the expression of hsa-miR-19b was negatively correlated with MYLIP expression in breast cancer patient samples. And the Pearson's correlation coefficient was −0.653, *p*= 0.0037 (Figure 1C, Supplementary Figure 1C). Additionally, we analyzed the expression level of MYLIP with the different status of breast cancer molecular classification markers (such as ER, PR and HER2). The results demonstrated that high expression level of MYLIP was positively related to the ER+, PR+ status, and negatively correlated with the ER-/PR-/HER2- (triple negative) status (Supplementary Figure 2A, 2B, 2D). However, its expression level didn't have significant difference between HER2+ and HER2- groups (Supplementary Figure 2C). These data hinted us that MYLIP might act as a protective factor in breast cancer development and indicate a relatively favorable prognostic effect during chemotherapies.

### miR-19b directly targets MYLIP gene and down-regulates its expression in breast cancer cells

**Figure 2:**
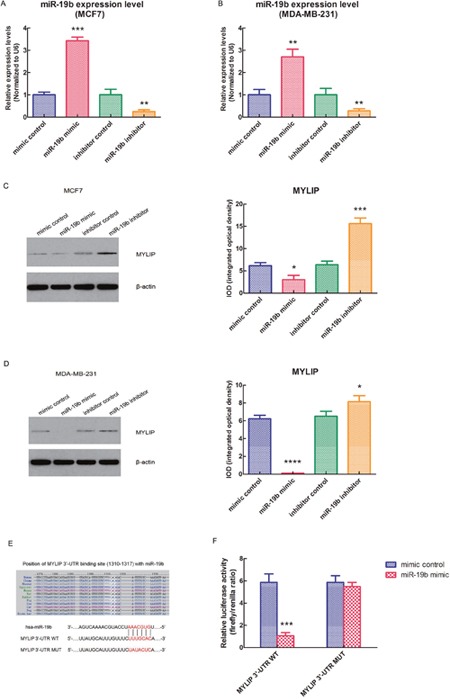
miR-19b directly targets MYLIP gene and down-regulates its expression in breast cancer cells **(A)** The relative expression levels of miR-19b in MCF7 cell line when transfected with miR-19b mimic, miR-19b inhibitor and their negative controls. The asterisks (**, ***) indicate a significant difference (*p* < 0.01, *p* < 0.001) respectively. **(B)** The relative expression levels of miR-19b in MDA-MB-231 cell line when transfected with miR-19b mimic, miR-19b inhibitor and their negative controls. The asterisks (**) indicate a significant difference (*p* < 0.01). **(C)** The protein expression levels of MYLIP and their integrated optical densities (IOD) in MCF7 cell line when transfected with miR-19b mimic, miR-19b inhibitor and their negative controls. The asterisk(s) (*, ***) indicate a significant difference (*p* < 0.05, *p* < 0.001) respectively. **(D)** The protein expression levels of MYLIP and their integrated optical densities (IOD) in MDA-MB-231 cell line when transfected with miR-19b mimic, miR-19b inhibitor and their negative controls. The asterisk(s) (*, ****) indicate a significant difference (*p* < 0.05, *p* < 0.0001) respectively. **(E)** The binding site of hsa-miR-19b with MYLIP 3′-UTR region (wild type, WT) and its mutant (MUT) sequences. **(F)** The relative luciferase activities of MYLIP 3′-UTR WT vector and MYLIP 3′-UTR MUT vector when co-transfected with miR-19b mimic and mimic control in HEK293 cell line. The asterisks (***) indicate a significant difference (*p* < 0.001).

In order to further verify that MYLIP is a direct target of miR-19b, we used the MCF7 and MDA-MB-231 breast cancer cell lines as models. First, we transfected these two cell lines with miR-19b mimic, miR-19b inhibitor and their negative controls. Then we tested the expression levels of miR-19b in these cell lines after the above treatments to confirm the miR-19b over-expression and knock-down efficiency (**p < 0.01, ***p < 0.001) (Figure 2A, 2B). Next, we detected the protein expression levels of MYLIP in MCF7 and MDA-MB-231 cell lines after transfected with miR-19b mimic, miR-19b inhibitor and their negative controls. And we screened and calculated the integrated optical densities (IOD) for the MYLIP bands. The results demonstrated that over-expression of miR-19b could significantly down-regulate the MYLIP protein expression. Conversely, inhibition of miR-19b expression could significantly up-regulate the MYLIP protein expression (*p < 0.05, ***p < 0.001, ****p < 0.0001) (Figure 2C, 2D). Furthermore, we constructed the MYLIP 3′-UTR WT (wild type) and MUT (mutant) vectors according to their binding sites with hsa-miR-19b seed sequences (highlighted in red) (Figure 2E), and co-transfected them with miR-19b mimic and mimic control in HEK293 cell line. Then we carried out the dual luciferase reporter assay and tested the relative luciferase activities through calculating the firefly/renilla ratios. The data hinted that the relative luciferase activity was significantly suppressed in the MYLIP 3′-UTR WT group compared with MYLIP 3′-UTR MUT group when transfected with miR-19b mimic (***p < 0.001) (Figure 2F), which suggested that miR-19b could directly regulate the expression of MYLIP gene through targeting its 3′-UTR region.

### miR-19b affects the expressions of cell adhesion molecules (E-Cadherin, ICAM-1, Integrin β1) and the Integrin β downstream signaling pathways

**Figure 3:**
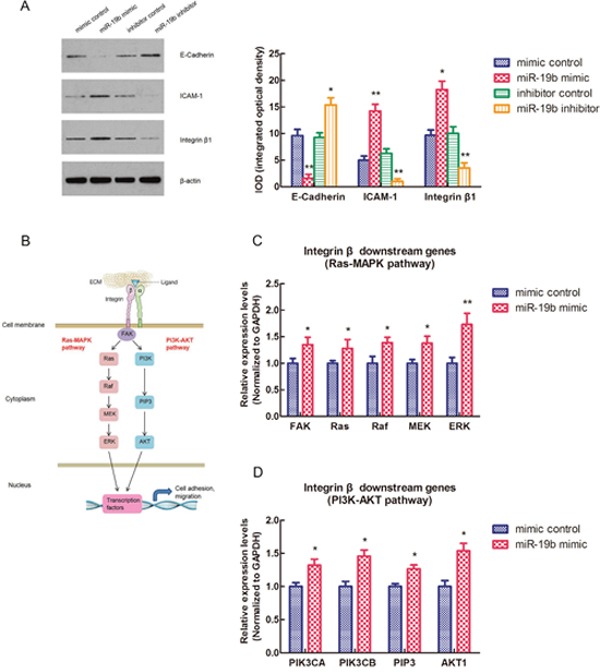
miR-19b affects the expressions of cell adhesion molecules (E-Cadherin, ICAM-1, Integrin β1) and the Integrin β downstream signaling pathways **(A)** The protein expression levels of E-Cadherin, ICAM-1, Integrin β1 and their integrated optical densities (IOD) in MCF7 cell line when transfected with miR-19b mimic, miR-19b inhibitor and their negative controls. The asterisk(s) (*, **) indicate a significant difference (*p* < 0.05, *p* < 0.01) respectively. **(B)** The schematic model for Integrin β downstream signaling pathways (Ras-MAPK pathway and PI3K-AKT pathway). **(C)** The relative expression levels of Ras-MAPK pathway genes in MCF7 cell line when transfected with miR-19b mimic and mimic control. The asterisk(s) (*, **) indicate a significant difference (*p* < 0.05, *p* < 0.01) respectively. **(D)** The relative expression levels of PI3K-AKT pathway genes in MCF7 cell line when transfected with miR-19b mimic and mimic control. The asterisk (*) indicates a significant difference (*p* < 0.05).

To investigate whether miR-19b could also affect the expressions of other cell adhesion molecules apart from MYLIP, we tested the protein expression levels of E-Cadherin, ICAM-1, and Integrin β1 in MCF7 cell line after transfected with miR-19b mimic, miR-19b inhibitor and their negative controls. And we screened and calculated the integrated optical densities (IOD) for the bands of these three molecules. The results showed that over-expression of miR-19b could significantly down-regulate the E-Cadherin expression level, but up-regulate the expression levels of ICAM-1 and Integrin β1. On the contrary, inhibition of miR-19b expression could significantly up-regulate the E-Cadherin expression level, but down-regulate the expression levels of ICAM-1 and Integrin β1 (*p < 0.05, **p < 0.01) (Figure 3A). Integrin β1 could activate two major downstream signaling pathways (Ras-MAPK pathway and PI3K-AKT pathway) in cytoplasm and plays a key role in cell adhesion and migration (Figure 3B). In order to further explore the effect of miR-19b on the Integrin β downstream signaling pathway genes, we conducted the RT-PCR experiment to check the relative expression levels of key genes in these two pathways in MCF7 cell line when transfected with miR-19b mimic and mimic control. The data indicated that over-expression of miR-19b could significantly up-regulate the expression levels of key genes in Ras-MAPK pathway (including FAK, Ras, Raf, MEK and ERK) and PI3K-AKT pathway (including PIK3CA, PIK3CB, PIP3 and AKT1) (*p < 0.05, **p < 0.01) (Figure 3C, 3D).

### Over-expression of miR-19b and inhibition of MYLIP facilitate the migration and metastasis of breast cancer cells

**Figure 4:**
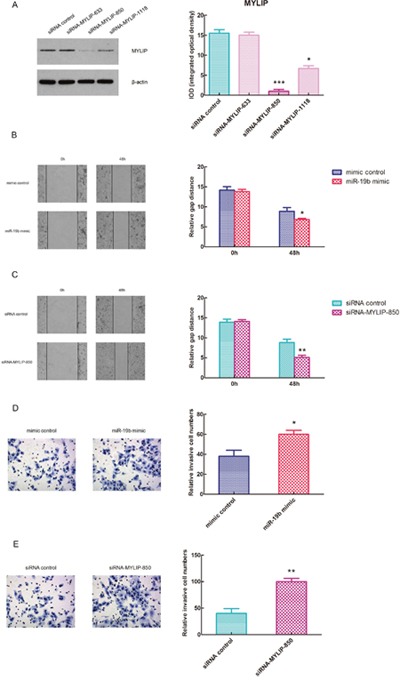
Over-expression of miR-19b and inhibition of MYLIP facilitate the migration and metastasis of breast cancer cells **(A)** The protein expression levels of MYLIP and their integrated optical densities (IOD) in MCF7 cell line when transfected with three different siRNA-MYLIP colonies and siRNA control. The asterisk(s) (*, ***) indicate a significant difference (*p* < 0.05, *p* < 0.001) respectively. **(B)** The wound healing assay for MCF7 cell line when transfected with miR-19b mimic and mimic control at 0h and 48h. The relative gap distances were calculated respectively. The asterisk (*) indicates a significant difference (*p* < 0.05). **(C)** The wound healing assay for MCF7 cell line when transfected with siRNA-MYLIP-850 and siRNA control at 0h and 48h. The relative gap distances were calculated respectively. The asterisks (**) indicate a significant difference (*p* < 0.01). **(D)** The transwell invasion assay for MCF7 cell line when transfected with miR-19b mimic and mimic control. The relative invasive cell numbers were calculated respectively. The asterisk (*) indicates a significant difference (*p* < 0.05). **(E)** The transwell invasion assay for MCF7 cell line when transfected with siRNA-MYLIP-850 and siRNA control. The relative invasive cell numbers were calculated respectively. The asterisks (**) indicate a significant difference (*p* < 0.01).

In order to effectively inhibit the expression of MYLIP, we adopted the siRNA strategy to construct three different siRNA-MYLIP colonies (siRNA-MYLIP-633, siRNA-MYLIP-850 and siRNA-MYLIP-1118). Then we transfected them with siRNA control into MCF7 cell line, and detected the protein expression levels of MYLIP and their integrated optical densities (IOD). The results demonstrated that siRNA-MYLIP-850 and siRNA-MYLIP-1118 colonies could suppress MYLIP expressions at different degrees, among them siRNA-MYLIP-850 had the most significant inhibition effect (*p < 0.05, ***p < 0.001) (Figure 4A). Then we conducted the wound healing assay for MCF7 cell line when transfected with miR-19b mimic/mimic control and siRNA-MYLIP-850/siRNA control, and measured their relative gap distances at 0h and 48h timepoints respectively. The data showed that over-expression of miR-19b and inhibition of MYLIP could significantly reduce the relative gap distances at 48h compared with the control groups (*p < 0.05, **p < 0.01) (Figure 4B, 4C), which facilitates the migration capacities of breast cancer cells. In the next step, we carried out the matrigel transwell invasion assay for MCF7 cell line when transfected with miR-19b mimic/mimic control and siRNA-MYLIP-850/siRNA control, and measured their relative invasive cell numbers respectively. The results indicated that over-expression of miR-19b and inhibition of MYLIP could significantly increase the relative invasive cell numbers compared with the control groups (*p < 0.05, **p < 0.01) (Figure 4D, 4E), which promotes the metastasis of breast cancer cells.

### miR-19b promotes breast tumor growth and affects the expressions of cell adhesion molecules *in vivo*

**Figure 5:**
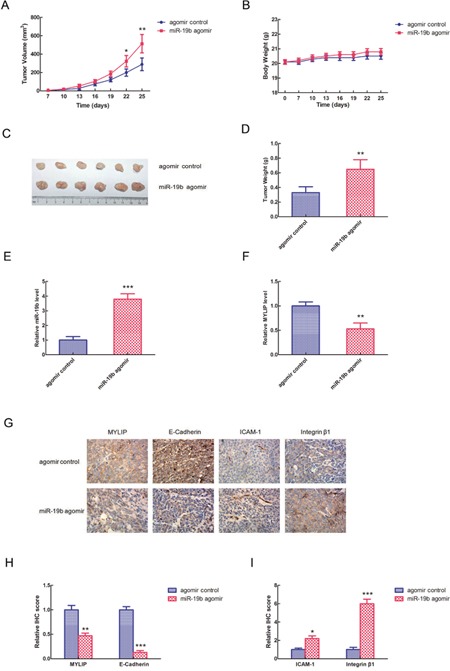
miR-19b promotes breast tumor growth and affects the expressions of cell adhesion molecules *in vivo* **(A)** The tumor volumes of BALB/c nude mice injected by MCF7 cells (1×10^6^) transfected with miR-19b agomir and agomir control respectively at different timepoints. The asterisk(s) (*, **) indicate a significant difference (*p* < 0.05, *p* < 0.01) respectively. **(B)** The body weights of BALB/c nude mice injected by MCF7 cells (1×10^6^) transfected with miR-19b agomir and agomir control respectively at different timepoints. **(C)** Photographs of breast tumors for miR-19b agomir and agomir control groups at 25 days of injection. **(D)** The relative tumor weights were measured for miR-19b agomir and agomir control groups. The asterisks (**) indicate a significant difference (*p* < 0.01). **(E)** The relative miR-19b expression levels in miR-19b agomir and agomir control groups. The asterisks (***) indicate a significant difference (*p* < 0.001). **(F)** The relative MYLIP expression levels in miR-19b agomir and agomir control groups. The asterisks (**) indicate a significant difference (*p* < 0.01). **(G)** The immunohistochemical (IHC) staining pictures for MYLIP and cell adhesion molecules (E-Cadherin, ICAM-1, Integrin β1) in miR-19b agomir and agomir control groups. **(H)** The relative IHC scores for MYLIP and E-Cadherin in miR-19b agomir and agomir control groups. The asterisks (**, ***) indicate a significant difference (*p* < 0.01, *p* < 0.001) respectively. **(I)** The relative IHC scores for ICAM-1 and Integrin β1 in miR-19b agomir and agomir control groups. The asterisk(s) (*, ***) indicate a significant difference (*p* < 0.05, *p* < 0.001) respectively.

To further validate the essential role of miR-19b in breast cancer development *in vivo*, we used the BALB/c nude mice as animal models, and injected them by MCF7 cells (1×10^6^) transfected with miR-19b agomir and agomir control respectively. 7 days after the injection, we measured the tumor volumes and body weights of BALB/c nude mice once three days until the 25 days, and drew the growth curves for miR-19b agomir group and agomir control group. The graphs revealed that the tumor volumes of miR-19b agomir group were significantly larger than agomir control group, especially at the 22 and 25 days (*p < 0.05, **p < 0.01) (Figure 5A), however, the body weights of these two groups didn't show significant differences (Figure 5B). These data hinted that miR-19b could effectively promote breast tumor growth *in vivo*, but didn't have much obvious effect on the body weights of nude mice. 25 days after the injection, we euthanized the mice of the two groups, separated the tumors, took pictures and weighed the tumor weights. The results showed that the tumors formed in miR-19b agomir group were much bigger than agomir control group (Figure 5C), and its tumor weights were significantly heavier than agomir control group (**p < 0.01) (Figure 5D). Then we extracted the RNA from the tumor tissues and tested the relative expression levels of miR-19b and MYLIP in miR-19b agomir group and agomir control group respectively. The date demonstrated that the expression level of miR-19b was significantly up-regulated in miR-19b agomir group (***p < 0.001) (Figure 5E), and the expression level of MYLIP was significantly down-regulated in miR-19b agomir group compared with agomir control group (**p < 0.01) (Figure 5F). In the next step, we conducted the immunohistochemical (IHC) staining for tumor samples to detect the expressions of MYLIP and cell adhesion molecules (E-Cadherin, ICAM-1 and Integrin β1) (Figure 5G) and let the pathologists to determine their relative IHC scores in miR-19b agomir and agomir control groups. The IHC scores suggested that the expression levels of MYLIP and E-Cadherin were significantly down-regulated, but the expression levels of ICAM-1 and Integrin β1 were significantly up-regulated in miR-19b agomir group compared with agomir control group (*p < 0.05, **p < 0.01, ***p < 0.001) (Figure 5H, 5I).

**Figure 6:**
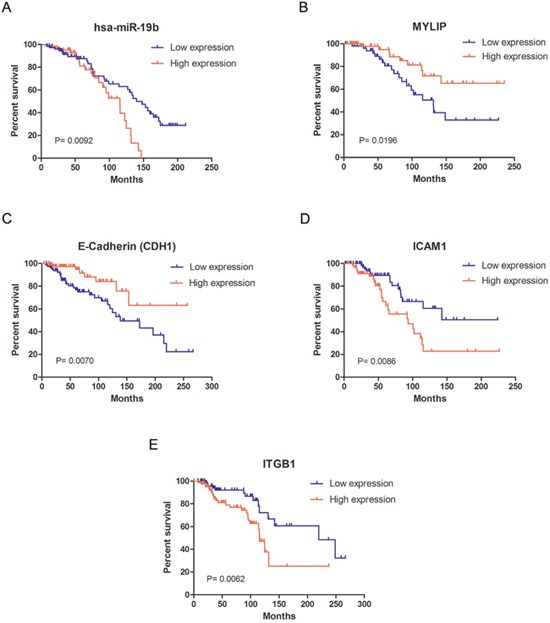
The survival curves for miR-19b, MYLIP and cell adhesion molecules in breast cancer patients **(A)** The survival curve of hsa-miR-19b in breast cancer patients, among them the low expression group (n=73) and high expression group (n=66). **(B)** The survival curve of MYLIP in breast cancer patients, among them the low expression group (n=56) and high expression group (n=59). **(C)** The survival curve of E-Cadherin (CDH1) in breast cancer patients, among them the low expression group (n=81) and high expression group (n=84). **(D)** The survival curve of ICAM-1 in breast cancer patients, among them the low expression group (n=76) and high expression group (n=63). **(E)** The survival curve of Integrin β1 (ITGB1) in breast cancer patients, among them the low expression group (n=82) and high expression group (n=87). All the primary expression data of these genes in breast cancer patients were downloaded from the TCGA database.

Additionally, using the primary expression data of breast cancer patients from the TCGA database, we drew the overall survival curves for hsa-miR-19b, MYLIP and cell adhesion molecules (E-Cadherin, ICAM-1 and Integrin β1) respectively. The curves indicated that the high expression levels of hsa-miR-19b, ICAM-1 and Integrin β1 (ITGB1) could significantly lower the survival rates for breast cancer patients compared with their low expression groups (*p* = 0.0092 for hsa-miR-19b curve, *p* = 0.0086 for ICAM-1 curve, *p* = 0.0062 for ITGB1 curve) (Figure 6A, 6D, 6E). The high expression levels of MYLIP and E-Cadherin (CDH1) could significantly elevate the survival rates for breast cancer patients compared with their low expression groups (*p* = 0.0196 for MYLIP curve, *p* = 0.0070 for CDH1 curve) (Figure 6B, 6C). These data hinted that hsa-miR-19b, ICAM-1 and Integrin β1 might be act as risk factors for breast cancer patients, while MYLIP and E-Cadherin could be considered as protective factors for breast cancer patients.

## DISCUSSION

**Figure 7:**
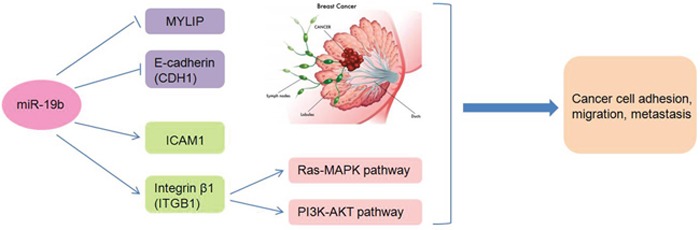
The schematic model of the regulatory mechanisms of miR-19b in breast cancer metastasis Through inhibiting the expressions of MYLIP and E-Cadherin, or up-regulating the expressions of ICAM-1 and Integrin β1 (accompanied by activating the Ras-MAPK and PI3K-AKT pathways), miR-19b exerts its essential roles in breast cancer cell adhesion, migration and metastasis.

In this article, we mainly illustrated the regulatory mechanisms of miR-19b promoting breast cancer metastasis through directly targeting MYLIP expression and affecting the expression levels of cell adhesion molecules (including E-Cadherin, ICAM-1 and Integrin β1) (Figure 7). Although numerous researches focused on breast cancer metastasis have already been carried out recently, the most striking findings and novelties for our study are that we verified the regulatory relationship between miR-19b and MYLIP, and testified their essential biological effects on breast cancer metastasis for the first time. Also we have built a close link between the cell adhesion molecules with cancer metastasis in breast tumorigenesis and development.

Cancer metastasis is a core issue and the most important topic in the cancer research field. Among all of the twelve cancer hallmarks, cancer metastasis take the leading role and decide the final fate for the cancer patients [17]. Once the metastasis occurred in distant organs, the survival rate and prognostic effect for cancer patients will be greatly reduced. The molecular events for metastasis are as follows: 1) the dysregulation of metastasis related genes, such as Ras, Mtsl, Nm23, TIAM-1, KAI1/CD82, RKIP, Kiss1, BRMS1, RhoGDI, MKK4, Drg-1 and so on [18–21]; 2) the abnormal expression of cell adhesion molecules, such as Integrins, Cadherins, Selectins, ICAM-1, VCAM-1, CD44 and so on [22–24]; 3) the abnormal expression of protein degrading enzymes, such as MMPs, t-PA, u-PA, Heparinase and so on [25]; 4) the over activation of tumor angiogenesis, and the key factors involved in this process including VEGF, bFGF, CD105 and so on [26]; 5) the imbalance of immune system states, and the major immune cells involved in anti-cancer immunity including NK cells, CTL cells, mononuclear macrophage cells, TIL cells and so on [27].

In our research, we mainly explored the downstream effects of miR-19b and its target MYLIP in breast cancer metastasis from the aspects of cell adhesion molecules. Through down-regulating E-Cadherin expression and up-regulating ICAM-1 and Integrin β1 expressions, miR-19b could obviously promote breast tumor growth and facilitate cancer metastasis. Actually, the biological functions and expression patterns of cell adhesion molecules in cancer metastasis are not the single-line model, but have the double-faceted characteristics. Take the Integrins for example, in the early stage of cancer development, the down-regulation of Integrins expressions will weaken the adhesions between tumor cells and basement membrane, so as to promote tumor growth and migration in the local region. Once the tumor cells infiltrate into the circulation, the high expression levels of Integrins will favor the adhesion of tumor cells into the vascular endothelial cells, and speed up the invasion and metastasis processes [28].

Moreover, for the crucial role of MYLIP and its relationship with the cell adhesion molecules in cancer metastasis, it is a quite new research field and much things still need to be done to reveal the real mystery under them. The recent studies have indicated that the polymorphism site of MYLIP p.N342S is associated with the response to lipid-lowering therapy and might be a pharmacogenetic marker in patients with familial hypercholesterolemia [29]. Besides, the p.N342S MYLIP polymorphism is also associated with high total cholesterol level and the modulation of MYLIP activity can affect LDL levels through increasing the degradation of LDL receptors. So the pharmacologic inhibition of MYLIP activity might become a useful method for the treatment of dyslipidemia [30]. All of the above results on MYLIP and lipid metabolism enlighten us novel insights to link the cancer metastasis with cancer metabolism, which includes the glucose metabolism, lipid metabolism, glutamine metabolism, amino acid metabolism and so on. Thus, apart from exploring the interactions between MYLIP and cell adhesion molecules contributing to cancer metastasis, the modulatory mechanisms of MYLIP in cancer metabolism are also worthwhile paying more attention in the cancer metastasis research field.

Last but not the least, the study for “star molecule” miRNA in carcinogenesis has been launched for many years. With the development of bioinformatics technologies, we can make the best use of multiple databases to compare miR-19b expression levels in different cancer types, predict their putative targets, set up the interaction networks for potential targets, analyze their downstream biological functions, and draw the survival curves to indicate their risk factors [31]. Furthermore, owing to the exosome research concepts and methods introduced into miRNA study field, it greatly expands the prospects of miRNA into the clinic application [32]. So in the next step, with the aid of exosome technologies, we can further explore miR-19b as a promising biomarker in the body fluid to assist tumor early detection and develop more of its downstream genes as therapeutic targets for breast cancer treatment. It will significantly facilitate the progression of translational medicine and elevate the diagnostic and therapeutic efficacy of breast cancer patients.

## MATERIALS AND METHODS

### Cell culture

The MCF7, MDA-MB-231 and HEK293 cell lines were cultured in DMEM with 10% fetal bovine serum (FBS), 1% glutamine, and 1% antibiotics. The cell lines were grown in a humidified incubator at 37°C with 5% CO_2_.

### miRNA and RNA extraction

Cellular miRNA extraction was performed using mirVana™ miRNA Isolation Kit (AM1560, Ambion) following the manufacturer's instructions. Total RNA extraction was performed using TRIzol Reagent (Invitrogen, Carlsbad, CA, USA) following the manufacturer's instructions.

### Real-time PCR assays

ThemiR-19b quantitative real-time PCR assays were performed using TaqMan® MicroRNA Assays (Applied Biosystems, USA), and U6 snRNA (Applied Biosystems, USA) was used as an internal control. For the MYLIP and Integrin β downstream genes quantitative real-time PCR assays, GAPDH was used as an internal control. And their expression levels were detected using SYBR Green I chemistry (Power SYBR Green PCR Master Mix, ABI Inc., USA). Real-time PCR and data collection were performed with an ABI 7500 sequence detection system. The relative expression levels were calculated using the 2^−ΔΔCt^ method.

### Western blot analysis

The total cell lysates were separated by SDS-PAGE gel and followed by Western blot. The images were processed and the integrated optical densities (IOD) of the bands were analyzed by Image Lab 4.0 (Bio-Rad Laboratories, Inc.) software packages. The antibodies used in this experiment were as follows: MYLIP (ab74562, Abcam, USA), E-Cadherin (ab76055, Abcam, USA), ICAM-1 (sc-390483, Santa Cruz, USA), Integrin β1 (ab155145, Abcam, USA) and β-actin (sc-47778, Santa Cruz, USA).

### Transient transfection

ThemiR-19b mimic, mimic control, miR-19b inhibitor, inhibitor control, siRNA-MYLIP-633, siRNA-MYLIP-850, siRNA-MYLIP-1118 and siRNA control were all synthesized by GeneChem, Shanghai, China. They were respectively transfected into MCF7 and MDA-MB-231 cells using Lipofectamine 2000 (Invitrogen, USA) according to the manufacturer's instructions.

### miRNA downstream targets prediction

The putative targets of miR-19b were predicted using the following six respective databases: miRecords (http://mirecords.biolead.org/), miRanda (http://www.microrna.org//miranda.html), TargetScan (http://genes.mit.edu/targetscan), PITA (http://genie.weizmann.ac.il/pubs/mir07/mir07_data.html), RNAhybrid (http://bibiserv.techfak.uni-bielefeld.de/rnahybrid/), and starBase (http://starbase.sysu.edu.cn/) [33].

### Luciferase reporter assays

3′-UTR sequences and the mutant sequences of MYLIP containing the putative miR-19b target sites were cloned in the plasmid SV40-Luc-MCS-pMIR-report-Vector (GeneChem, Shanghai, China). Luciferase reporter assays were performed in HEK293 cells using the Dual Luciferase Reporter Assay Kit (Cat#: E1910, Promega, USA). Firefly luciferase values were normalized to Renilla, and the ratios of Firefly/Renilla activity were presented.

### Wound healing assay

When the cells were grown to 90% confluence after the transfection, a straight scratch in the cell monolayer was created by a 10μl pipette tip. Images of the scratched area (wound) were taken at the indicated time points under a microscope [34].

### Transwell invasion assay

Before cell seeding, 24-well transwell chambers (8μm pores; Corning, NY, USA) were coated with matrigel matrix (BD Biosciences, CA, USA). 5×10^4^ transfected MCF7 cells suspended in 200μl DMEM (with 1% FBS) were seeded into each chamber. DMEM medium with 15% FBS was placed in the 24-well's bottom wells. Then the cells were allowed to migrate for 48h at 37°C. After that the chambers were fixed with 10% methanol for 15min and invasive cells were stained with 2% crystal violet for 5min. The stained cells were counted under a microscope [35].

### *In vivo* tumorigenesis

Six- to eight-week-old female BALB/c nude mice (purchased from Beijing Vital River Laboratory Animal Technology, Beijing, China) were used in this experiment and randomly divided into two groups for six mice each. The 0.72 mg/60days slow release estradiol pellets (purchased from Innovative Research of America, FL, USA) were adopted to give the oestrogen supplement for supporting the growth of MCF7 cells in nude mice. The pellets were implanted subcutaneously into the dorsal flank of female BALB/c nude mice. After BALB/c nude mice were anaesthetized and the skins were incised, MCF7 cells (1×10^6^) transfected with miR-19b agomir and agomir control (Ribobio, Guangzhou, China) in 20μl phosphate-buffered saline mixed with 1:1 ratio growth factor reduced Matrigel (BD Biosciences, CA, USA) were orthotopically injected into mammary fat pads. 7 days after the injection, the tumor volumes and body weights of BALB/c nude mice were measured once three days until the 25 days. Tumor growth was monitored over time using electronic calipers. The greatest longitudinal diameter (length) and the greatest transverse diameter (width) were measured. Tumor volumes were calculated by the modified ellipsoidal formula: volume = 1/2(length x width^2^). Then the mice were euthanized and the tumors were separated to weigh their weights. After that the tumors were fixed with phosphate-buffered neutral formalin and embedded by paraffin for IHC staining. All animal procedures were performed in accordance with institutional guidelines.

### Immunohistochemistry (IHC) and scoring

The tissues were sectioned, treated with 3% H_2_O_2_, and then incubated in 5% goat antiserum. Non-serial tissue sections were incubated with the primary antibodies against MYLIP (1:100; Abcam, USA), E-Cadherin (1:200; Abcam, USA), ICAM-1 (1:200; Santa Cruz, USA), Integrin β1 (1:100; Abcam, USA) overnight, and then with biotin-labeled secondary antibodies. Streptavidin-peroxidase complex was added, and the sections were stained with 3,3′-diaminobenzidine (Maixin Biotech, Fuzhou, China) prior to microscopy analyses. All sections were independently scored by three experienced pathologists. Scoring was based on the percentages of positive cells with different staining intensities [36].

### Bioinformatical analysis of miRNA and genes expression levels

The expression levels of hsa-miR-19b and MYLIP in breast cancer samples versus their adjacent normal breast tissues were analyzed by The Cancer Genome Atlas (TCGA) database (https://cancergenome.nih.gov/) and starBase database (http://starbase.sysu.edu.cn/). The primary expression data for drawing the survival curves ofhsa-miR-19b, MYLIP and cell adhesion molecules (E-Cadherin, ICAM-1 and Integrin β1) in breast cancer patients, were downloaded from the TCGA database, SurvExpress database (http://bioinformatica.mty.itesm.mx:8080/Biomatec/SurvivaX.jsp), SurvMicro database (http://bioinformatica.mty.itesm.mx:8080/Biomatec/Survmicro.jsp) and Kaplan Meier-Plotter database (http://kmplot.com/analysis/).

### Statistical analysis

All quantitative data were expressed as mean values ± S.D. of at least 3 independent experiments. Statistical analysis was performed using SPSS17.0. Significant differences between two groups were compared using the Student's t-test, and comparisons among more than two groups were performed using analysis of variance (ANOVA). The correlation analysis was conducted using Pearson's correlation coefficient. *p*-values < 0.05 were considered to be statistically significant.

## SUPPLEMENTARY MATERIALS FIGURES


