# Supplementary material for: miR-19b promotes breast cancer metastasis through targeting MYLIP and its related cell adhesion molecules
Source: Oncotarget. 2017 Jul 17;8(38):64330–43. doi: 10.18632/oncotarget.19278 (PMC5610006; doi:10.18632/oncotarget.19278)
Supplement: Supplementary file 1 [file oncotarget-08-64330-s001.pdf]

miR-19b promotes breast cancer metastasis through targeting MYLIP and its related cell adhesion molecules

SUPPLEMENTARY MATERIALS

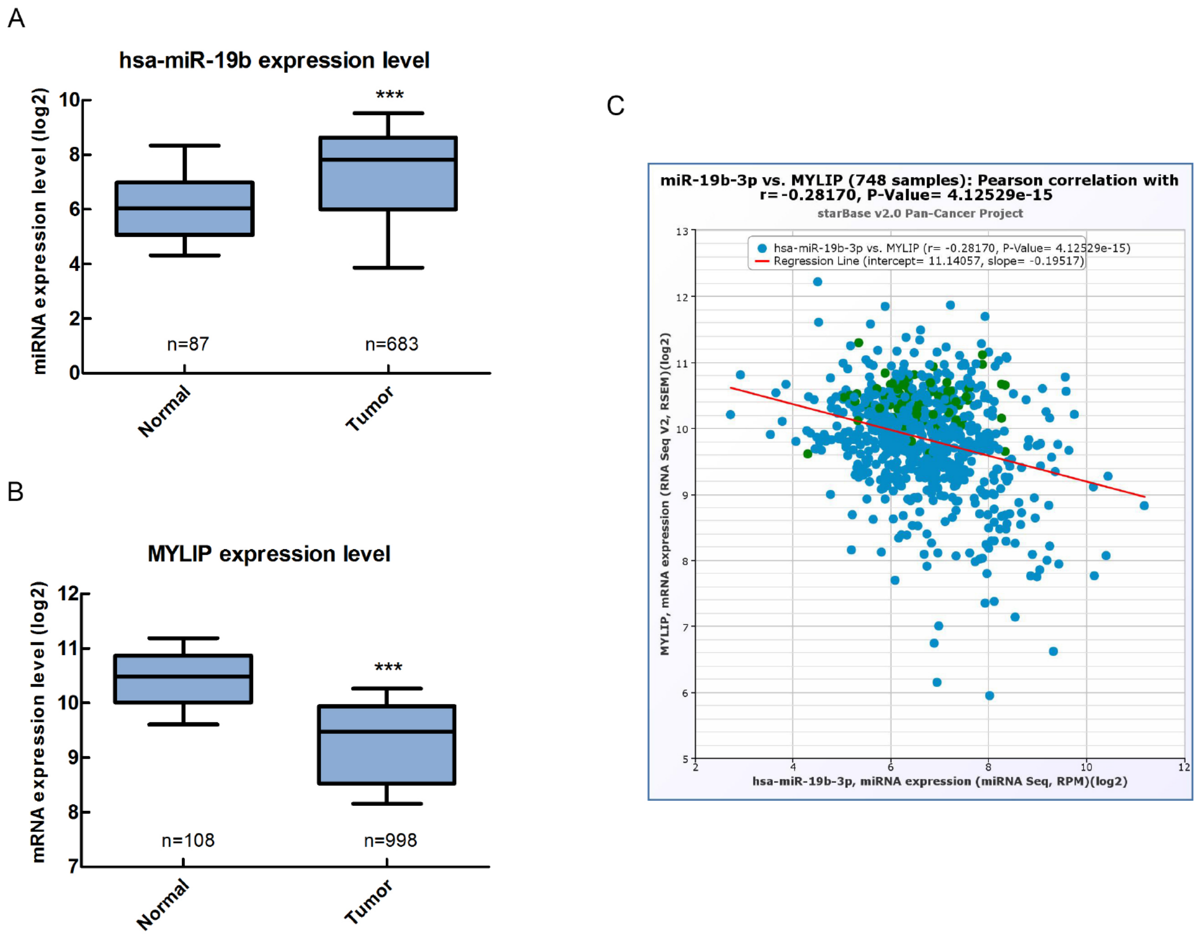

Supplementary Figure 1: The expression levels of hsa-miR-19b and MYLIP in breast cancer samples and their adjacent normal tissues, and the negative correlations between hsa-miR-19b and MYLIP in breast cancer patient samples. (A) The hsa-miR-19b expression level in breast cancer samples (n=683) and adjacent normal breast tissues (n=87). The primary hsa-miR-19b expression data (log2) in breast cancer patients were downloaded from the TCGA database. The asterisks (***) indicate a significant difference ($p < 0.001$). **(B)** The MYLIP expression level in breast cancer samples (n=998) and adjacent normal breast tissues (n=108). The primary MYLIP mRNA expression data (log2) in breast cancer patients were downloaded from the TCGA database. The asterisks (***) indicate a significant difference ($p < 0.001$). **(C)** The correlation between hsa-miR-19b and MYLIP in breast cancer samples (n=748). The figure was downloaded from the starBase database and all the primary hsa-miR-19b expression data (log2) and MYLIP mRNA expression data (log2) in breast cancer patients were analyzed by the TCGA database.

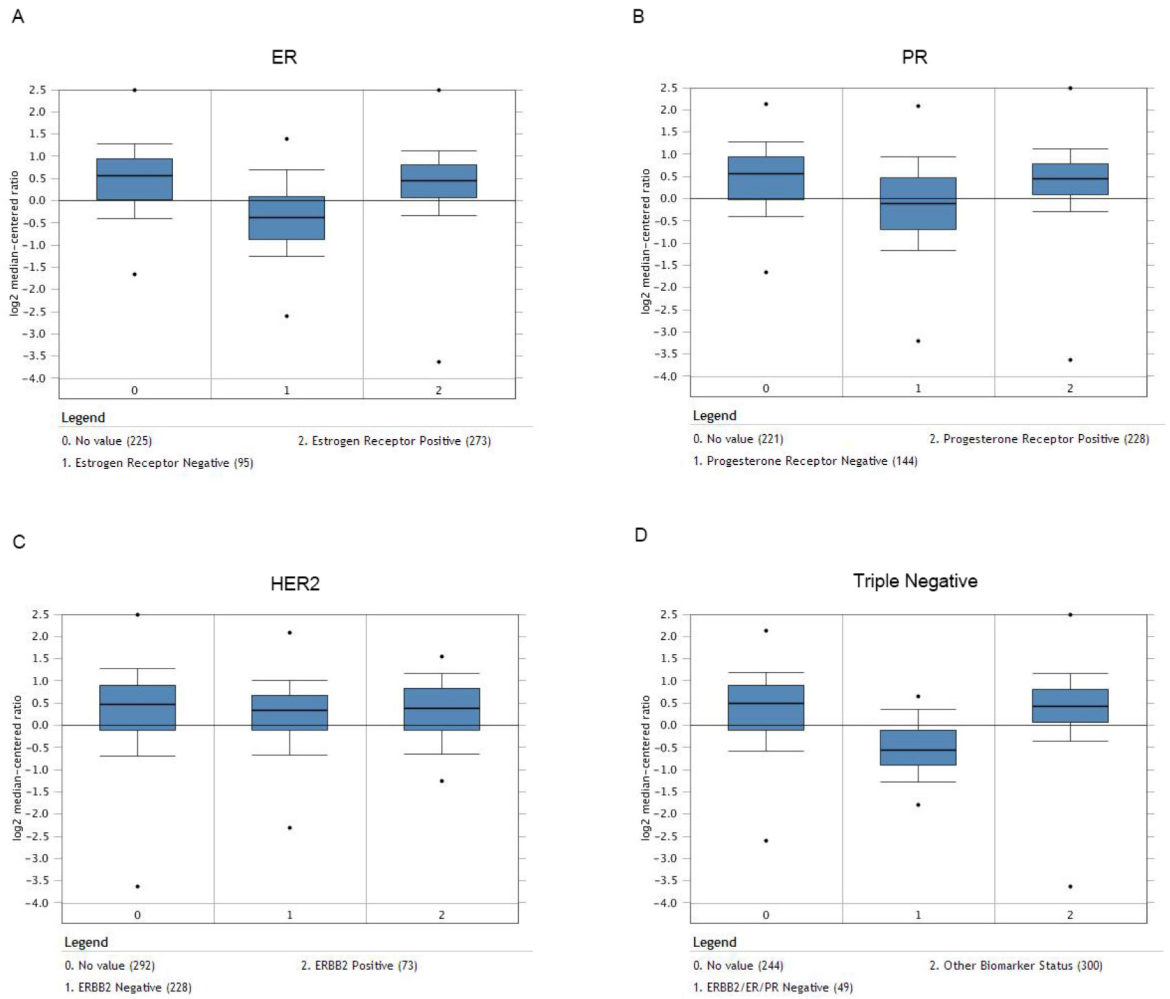

Supplementary Figure 2: The expression level of MYLIP was associated with the status of breast cancer molecular classification markers. **(A)** The expression level of MYLIP in Estrogen Receptor (ER) positive and negative breast cancer samples. **(B)** The expression level of MYLIP in Progesterone Receptor (PR) positive and negative breast cancer samples. **(C)** The expression level of MYLIP in ERBB2 (HER2) positive and negative breast cancer samples. **(D)** The expression level of MYLIP in ERBB2/ER/PR negative (Triple negative) breast cancer samples. The figures were downloaded from the Oncomine database and all the primary MYLIP mRNA expression data (log2) in breast cancer patients were analyzed by the TCGA database.
